# Notes on the Physiological and Therapeutic Effects of the Chloride of Ammonium

**Published:** 1856-01

**Authors:** Alexander Lindsay


*Notes on the Physiological and Therapeutic Effects of the Chloride of Ammonium.* By ALEXANDER LINDSAY, M. D.

It has been frequently remarked that our Materia Medica is sufficiently extended. Substances have doubtless been included amongst our medicines that perhaps ought not to have been accounted such. Yet it is as likely that many that have had a merely passing reputation —that have been named only to be left unheeded—may, nevertheless, not be so useless as their neglect would seem to indicate. How this should happen it is unnecessary to inquire. It is probable, however, that, in many cases, a lauded remedy is trusted, without, at the same time, attending to those general indications which an enlightened treatment would direct to be conjoined. This is no mere supposition; and thus it is that the therapeutic effect of even a useful medicine may be completely lost. In the treatment of chronic disease, the practitioner is often disappointed in the action of medicines, even with those that a large experience has shown to be useful. The causes that lead to this result are often difficult to discover. They may reside in the medicine; more frequently, however, they depend on peculiarities in the constitution of the patient. In such cases, a change of remedy is often attended with the happiest results, and hence the advantage of having at our command medicines whose actions are allied.

I have made these remarks preliminary to the urging on the notice of the profession an important medicinal agent, already included in our Materia Medica. I refer to the chloride of ammonium ($NH_4$ ), *muriate of ammonia*, or *sal ammoniac*, as it has been variously named. Internally it is seldom prescribed in British practice, although its external employment is not so rare. On the Continent it is otherwise. In France, but particularly in Germany, writers on the Materia Medica inform us that its internal administration is frequent. The knowledge of this fact induced me, some years since, to give it a trial, and since then I have recommended it to the attention of others. My experience of its advantages, conjoined with that of others who acted on my suggestion, have prompted the endeavor to secure for it a more general trial.

Iodine, bromine, and chlorine are readily distinguished the one from the other; yet, in some respects, they are physically allied, and they are so closely related chemically, that they have been grouped as a family. Further, there is a marked similarity as regards the therapeutic effects of the salts, resulting from their union with metals. These considerations, coupled with the assumed nature of the basyl ammonium, would naturally lead to the conclusion that the action of the chloride, in diseased states, would be of a character similar and equal to others held in higher repute; my own experience of the salt has shown such to be the case.

It may be useful to refer briefly to the physiological actions of the medicine under consideration. A knowledge of these cannot, it is true, serve as a guide to its employment in disease, yet it will enable us to measure its influence on the system, and to conclude in a general manner as to its therapeutic action. For where a physiological change in any given case is not observed, but little alteration is to be expected in morbid conditions.

The information possessed regarding the actions of the chloride on the healthy body is very limited. Pereira's Elements of Materia Medica contains all I have been able to glean on the subject. The statements there, however, refer to large doses; that author remarks as follows:—Its local action is irritant. Its chemical influence is not very obvious. It dissolves mucus, but does not coagulate albumen. Weber tried the salt on himself. He took from ten to twenty grains for a dose, which he repeated at the end of an hour. The effects were a sensation of warmth and oppression in the stomach, headache, and increased desire for passing urine." So far as I am aware, the influence of medicinal doses, continued for a certain time in healthy individuals, has not been recorded. I was anxious to ascertain this, and have been aided in the inquiry by two intelligent pupils, who willingly agreed to subject themselves, along with me, to the action of the chloride. Daily, for a week previous to the experiment, the state of the appetite, the nature and amount of the food, the condition of the bowels, the frequency of the pulse, with the amount and density of the urinary secretions, were carefully noted. The medicine was then taken for a week, and similar observations recorded. The amount taken was in one case 18 grains per day, a second 13½ grains, and the third 9 grains. These quantities were divided into three equal doses, and were swallowed dissolved in two ounces of water. No comparison of the results was made till the observations were concluded. The following is a brief summary of these, from the notes now before me:—

On the second day after beginning the medicine, a buoyancy of the system was experienced that rendered ordinary pursuits a pleasure, and fitted body and mind for increased exertion. The uniformity of this result was the more remarkable as the experimenters represent types of the nervous, sanguineous, and lymphatic temperaments respectively. The feeling was least developed in the last. He employed the smallest dose. In all, the appetite was much improved. Where the smallest quantity of the salt was taken, the amount of food was doubled. The feculent discharges were in all much augmented. The mucous follicles of the intestinal tube seemed to be stimulated to a much increased secretion. In two, the force and frequency of the heart's action were diminished. The rate of the pulse in the gentleman employing the smallest dose was accelerated. In all, the chloride increased the urinary secretion. It cannot, however, be classed as a renal hydragogue. The increase of fluid ranged from six to twelve ounces in the twenty-four hours. In the two cases, where the largest and smallest doses were used, it acted as a renal depurant, the excess of solids varying from 70 to 160 grains daily. In the other no change in this respect was noticed; but it may be necessary to remark, that the effect on the bowels appeared to be greatest in the individual making use of the medium dose.

The cases in which I have employed the chloride of ammonium in practice have been limited to chronic diseases, such as result from inflammatory action, or where there is a local ailment existing as the expression of a dyscrasial condition. Particularly, I have prescribed the salt in cases of chronic bronchitis, in enlargement of the lymphatic glands, whether resulting from scrofulous disease or dependent on a syphilitic taint, in chronic skin diseases, and in cases of chronic rheumatism.

The chloride has been, I may remark, exhibited also in acute diseases; in some forms of fever, and in the milder cases of pneumonia. Further, Dr. Watson, in his lectures, has testified as to its efficacy in certain forms of facial neuralgia; and Dr. Ebden, in the Indian Annals of Medical Science for April, 1854, states that it is a powerful and valuable remedy for the relief of neuralgic pain generally. He writes—"In facial neuralgia, tic douloureux, nervous headache and toothache, not excepting sciatica, and even in one case of neuralgic dysmenorrhœa, I have often given it, and been convinced, after a full trial, of its merits."

Of its therapeutic effects in acute diseases, I have no knowledge, nor have I prescribed it in neuralgic affections; but, from what I have seen of its physiological action in small doses, I can readily believe that the quantities prescribed by Dr. Ebden must have had an important influence.

I commenced the use of the salt in morbid conditions of the pulmonary mucous membrane, where the exuded (mucus) secretion was tough and tenacious. In such cases, its action was often remarkable, the exudate becoming speedily altered in quality and consistency. It has probably been from observing this effect that some have attributed to the chloride of ammonium qualities similar to the mercurial preparations. As liquefacients their influence is well known. On this account they are frequently employed. But in cases in which their use is contra-indicated, as frequently happens in chronic bronchitis, I can confidently recommend that their place be supplied by the preparation under consideration, certain that if the case be well chosen, the benefit will soon be apparent, and equally certain that no prejudicial result will follow its employment. In this respect it differs from the alkalies or their carbonates, the prolonged employment of these disordering the digestive and assimilative functions, and ultimately producing a condition similar to scurvy, the nutrition of the body generally becoming impaired. It is very different with the chloride, its long-continued use never giving rise to symptoms of general cachexia. The testimony of many observers agree in this.

The efficiency of the ammonium salt, as a remedy in the cases noted, led me gradually to extend its use, and subsequent trials, frequently repeated, have shown that in cases of chronic rheumatism it often proves of great advantage. It is doubtless true we will be occasionally disappointed. This, however, happens often with our best remedies; but in those forms of rheumatic disease in which there is but little constitutional disturbance—those cases, in short, in which the iodide of potassium is found advantageous—the chloride may be given with every likelihood of benefit. In this affection, I have given the salt a very extended trial; but, in recommending its use, I need scarcely hint that it is not to be depended on to the exclusion of those secondary means—warmth, frictions, &c., that form a part of any treatment we may employ. I have also tried the chloride in periosteal inflammation of chronic character, and having a syphilitic origin. Here its advantagea are not so apparent, yet it seldom fails to give relief, and in cases wheres the iodide of potassium had ceased to exert any apparent influence on the disease, the substitution of the chloride has been followed by a rapid cure.

In enlargement of the lymphatic glands, I have not had such an experience of the use of the chloride as enables me to speak with confidence of its action. Yet more or less benefit will follow its prolonged employment. In that variety of bubo aptly designated Indolent, which so often exhausts all ordinary remedies, and frequently our patience, the use of the chloride often speedily effects its removal. I am in the habit here of applying it also externally. A strong solution being employed (ʒii. to the ℥i.), lint is soaked in this, applied to the swollen surface, and covered with oiled silk. In such cases, the general treatment must not be forgotten. The state of the bowels must be watched, and, if need be, corrected, the diet employed being nutritious. Carefully-applied pressure over the swelling is also a valuable auxiliary.

In another point of view, the chloride of ammonium has an advantage. It is cheap. This precludes the likelihood of intentional adulteration. Sometimes it is impregnated with iron, and, it is said, occasionally with lead. As regards the former, when present, it exists in such small quantity that it can in no way interfere with its action; and as regards the latter, any samples I have tested showed no evidence of its presence.

By writers on the Materia Medica, the dose of the chloride is stated to be from 5 to 30 grains. The quantity prescribed by me has varied from 5 to 10 grains, three or four times a day. Dr. Ebden, in the paper already noticed, states he employs from 25 to 35 grains in neuralgic affections, repeated at short intervals. I have never given the salt in such doses. His experience, however, accords with my own as to the effect on the system of the quantities I am in the habit of administering. This agreement was to me the more gratifying, as I was not aware of the existence of his paper till these remarks were nearly completed, and it adds to the confidence I have in urging the medicine on professional attention.

The chloride of ammonium may be administered in simple or medicated waters. Where there is evidence of febrile disturbance, small doses of emetic tartar may be advantageously added. I frequently prescribe it with some bitter infusion, as quassia, cascarilla, gentian, &c. In cases where an anodyne may be necessary, the solution of the muriate of morphia may be conjoined in appropriate doses. It need scarcely be added, that the alkalies with their carbonates, as also the nitric and sulphuric acids, are incompatible.

The preceding observations have been limited to a mere narrative of observed results. Any endeavor to explain the nature of the influence exercised by the chloride over the organism has been carefully avoided. To judge of the action or effect of a medicine is always sufficiently difficult, without attempting to accomplish more. Even where the mind is least biassed, the accidental is often apt to be confounded with the essential, and antecedents linked with results with which they may be but very remotely related. This is not to be wondered at when we think on the very varied influences that combine to modify disease, and, as a necessary sequence, the actions of those agents we employ for its removal. Of disease we know nothing other than what is made known by symptoms and observed structural change, and of medicines only so much as they alter or modify these. Frequently the mind is not content with this, seeking to penetrate further, leaving the true field of observation, and attempting to grasp at what is placed far beyond mental reach. Thus it is that the science of therapeutics has not advanced with that steady onward step which has marked the progress of other departments of knowledge. The properties of matter are studied, the circumstances that modify these observed, and the information so acquired is applied to the purposes of life. The day is past in which it was sought to elicit its ultimate nature or essence. So ought it to be with medicines. Their actions, and the circumstances that modify these, should alone be investigated. The therapeutic power of the mercurial compounds is acknowledged, and every day applied to the treatment of disease; yet how little, how very little, of useful knowledge is to be gleaned by the study of the various hypotheses that have been offered to explain their action.

These remarks may be considered by some foreign to the subject of this paper. They will, however, serve to show why I have not saw fit to attempt to explain the *modus operandi* of the chloride. Of this I know nothing. I have seen its beneficial employment, and presume to think that, when in more general use, it will be assigned a position amongst our more valuable alterative, resolvent, and liquefacient remedies.

The following cases are appended in illustration of the effects of the salt. Numerous others might easily have been added:—

*Jan.* 25, 1855.—*J. W., hawker, aged* 45.—Has suffered from winter cough for many years. Four days ago was much exposed to wet, and since been unable to leave bed. When seen, skin hot, tongue furred, urine scanty, and bowels constipated. Pulse 120. Severe cough; scanty expectoration, and complains much of a feeling of constriction of the chest. The stethoscope afforded the physical signs usual in bronchitis. Ordered him to be purged, afterwards to have *tart. antimon. et potassæ*, in nauseating doses, and a mustard poultice to chest.

*Feb.* 2*d.*—Feverish symptoms gone. Cough still very severe. Complains of great difficulty of getting up the expectoration, which is so tenacious that he has to withdraw it from the mouth by the fingers. Ordered a teaspoonful of the following mixture, four times a day, in water:—

℞ Chloridii Ammonii, - - - - ʒij.

Sol. Mur. Morph., - - - ʒiis.

Syr. Simplicis, - - - - - ʒvj.

Aq. Puræ, - - - - ℥iv. Solve.

Within twenty-four hours a marked improvement began, which continued till the expectoration acquired an almost watery consistency. Ten days after he was in his ordinary health.

*August* 15, 1855.—*Mrs. G., aged* 66.—Has been complaining of cough for some weeks, with slight difficulty of breathing. Is able to move about for the greater part of the day, but complains of weakness and pain, referred to anterior and lower part of chest. Pulse 70. Expectoration scanty and tenacious. Bowels had been freely purged before I saw her. Dry mucous rales heard over upper part of chest. Ordered the foregoing mixture, without the solution of the muriate of morphia.

27*th.*—Has still a little cough. Sputa came up freely. Is gaining strength. Has now no pain in chest, and the appetite is much improved.

*July* 1, 1855.—Mrs.———, married, and has had four children. Contracted syphilis, six months ago, from her husband. Was mercurialized at the time by a surgeon in the country, and used afterwards, so far as I could learn, iodide of potass. She came under my care suffering from constitutional disease, manifesting itself on the skin, with sore throat and severe pains in the limbs. Her appearance was extremely unhealthy. Appetite nearly gone. Bowels disordered.

After touching the throat with the nitrate of silver, and attending to the bowels, she was ordered to sponge the body, night and morning, with a tepid saturated solution of common salt, and to have a teaspoonful of the following mixture three times a day:—

℞ Chloridii Ammonii, - - ʒiiss.

Acid-Hydro-Chlor., dil., - - ʒiss.

Aq. Puræ, - - - ℥ivss. Solve.

Shortly after beginning the medicine she began to improve, and at the end of four weeks all the symptoms had nearly disappeared.—*Glasgow Med. Jour.*

